# An updated systematic review and meta-analysis of laparoscopic versus ultrasound-guided transversus abdominis plane block in laparoscopic colorectal surgery

**DOI:** 10.3389/fmed.2026.1790253

**Published:** 2026-04-24

**Authors:** Yujie Zhang, Xianfang Hu, Shulu Li, Jikai Ni, Kun Zhang

**Affiliations:** 1 Linyi Hospital of Traditional Chinese Medicine, Linyi, Shandong, China; 2 Shandong University of Traditional Chinese Medicine, Jinan, Shandong, China; 3 Shenzhen Hospital (Fu Tian) of Guangzhou University of Chinese Medicine, Shenzhen, Guangdong, China

**Keywords:** colorectal surgery, laparoscopic, meta-analysis, transversus abdominis plane block, ultrasound

## Abstract

**Background:**

Transversus abdominis plane block (TAPB) has become a fundamental component of multimodal analgesia for laparoscopic colorectal surgery. Previous meta-analyses comparing laparoscopic-guided TAPB (Lap-TAPB) with ultrasound-guided TAPB (US-TAPB) were constrained by small sample sizes and lacked integration of emerging evidence from recent randomized controlled trials (RCTs). Given the increasing number of studies published since 2023, an updated review is warranted.

**Objectives:**

To conduct an updated systematic review and meta-analysis comparing Lap-TAPB with US-TAPB in terms of analgesic efficacy and perioperative outcomes in patients undergoing laparoscopic colorectal surgery.

**Methods:**

PubMed, Embase, and Web of Science were searched, from their inception until November 2025, for studies evaluating Lap-TAPB versus US-TAPB and reporting postoperative analgesic or clinical outcomes following the PRISMA guidelines. The primary outcome was 24-h postoperative opioid consumption, whereas the secondary outcomes included pain scores at 24 h (at rest), postoperative nausea and vomiting (PONV), operative time and complications.

**Results:**

Five studies involving 585 patients were included in this review. No significant differences were observed in 24-h postoperative opioid consumption with Lap-TAPB (standardized mean difference (SMD) −0.16, 95% confidence interval (CI) = −0.39 to 0.08, *p* = 0.20), pain scores at rest at 24 h (SMD −0.17, 95% CI = −0.39 to 0.04, *p* = 0.12), incidence of PONV (odds ratio (OR) = 0.97, 95% CI = 0.36–2.65, *p* = 0.96), operative time (SMD 0.05, 95% CI = −0.19 to 0.30, *p* = 0.67), and complications (OR = 1.25, 95% CI = 0.77–2.03, *p* = 0.37).

**Conclusion:**

Lap-TAPB did not result in significantly lower 24-h postoperative opioid consumption, pain scores at 24 h (at rest), PONV incidence, operative time and complications compared to US-TAPB. However, it eliminates the need for ultrasound devices while decreasing the logistical complexity of the procedure.

## 1 Introduction

Enhanced Recovery After Surgery (ERAS) protocols, which are widely adopted across high-volume centers globally, emphasize multimodal, opioid-sparing analgesia to accelerate gastrointestinal recovery, facilitate early mobilization, and minimize postoperative complications, such as ileus and nausea (1). Laparoscopic colorectal surgery has become the standard of care for a wide range of benign and malignant colorectal diseases (2).

Within this framework, the transversus abdominis plane block (TAPB) has become a fundamental component of perioperative pain management. It was initially described by Rafi in 2001 and has subsequently been shown to provide effective postoperative analgesia following major abdominal surgery in a randomized setting (3). TAPB, as a type of local anaesthetic technique, involves the injection of a local anesthetic between the transversus abdominis and internal oblique muscles to allow infiltration of the segmental nerves at the T8-L1 level. Subsequent randomized controlled trials (RCTs) have shown that TAPB provides effective somatic analgesia to the anterior abdominal wall and markedly reduces postoperative opioid requirements following major abdominal procedures (4, 5). In laparoscopic colorectal surgery, TAPB has been associated with improved pain control, earlier restoration of bowel activity, and shorter hospital stays (6, 7).

Two principal techniques are currently employed to deliver TAPB, namely, ultrasound-guided (US-TAPB) and laparoscopic-guided (Lap-TAPB). US-TAPB, typically performed by anesthesiologists, allows real-time visualization of fascial layers and needle trajectory, thereby enhancing the accuracy and reducing the risk of intraperitoneal injection. Contrarily, Lap-TAPB is performed by the operating surgeon under direct laparoscopic visualization at the end of the procedure to see the successful deposition of the local anesthetic in the target plane. Despite the increasing use of both modalities, their comparative effectiveness remains unclear. A recent meta-analysis of three trials revealed significantly lower opioid consumption and pain scores with Lap-TAPB but no differences in operative time, postoperative nausea and vomiting (PONV) incidence, or complication rates (8).

Since 2023, several new studies have investigated the effects of different TAPB protocols on the postoperative outcomes of laparoscopic colorectal surgery, resulting in different conclusions. Therefore, this study aimed to conduct a comprehensive systematic review and meta-analysis in accordance with the Preferred Reporting Items for Systematic Reviews and Meta-Analyses (PRISMA) guidelines so as to compare the efficacy of Lap TAPB and US-TAPB in laparoscopic colorectal surgery.

## 2 Methods

### Study design and protocol registration

This systematic review and meta-analysis was conducted following the PRISMA guidelines (9); however, ethical approval or informed consent was not required. Although not formally registered in PROSPERO, the structure and methodology closely adhere to PROSPERO standards, including the prespecification of the inclusion criteria, outcomes, and review methods.

### Eligibility criteria

The eligibility criteria were established using the PICOS (Population, Intervention, Comparison, Outcome, Study) framework:

#### Population (P)

Adult patients undergoing laparoscopic colorectal surgery for benign or malignant conditions.

#### Intervention (I)

Lap-TAPB, defined as any TAPB performed intraoperatively under direct laparoscopic visualization.

#### Comparator (C)

US-TAPB, performed preoperatively or intraoperatively by an anesthesiologist using real-time ultrasonography.

#### Outcomes (O)

At least one postoperative analgesic or perioperative clinical outcome:

Postoperative opioid consumption within the first 24 h, postoperative pain scores at rest (24 h), PONV, operative time and complications.

#### Study design (S)

RCTs, retrospective studies, case series, reviews, editorials, and noncomparative studies were excluded.

### Search strategy

A comprehensive literature search was conducted on PubMed, Embase, and Web of Science from their inception until November 26, 2025. No language restrictions were applied. The search strategy involved the use of combinations of the following Medical Subject Headings and related keywords: [“Transversus abdominis plane block” OR “TAPB” OR “Block”] AND [“Colon” OR “Rectal” OR “Colorectal”] AND [“Laparoscopic”].

Two reviewers (XFH and SLL) independently screened the titles and abstracts of all the identified citations. Potentially eligible studies were subjected to full-text screening. In addition, the reference lists of all the included studies were manually searched to identify any further relevant publications that may have been missed by the electronic search.

### Data collection

Data were extracted independently by YJZ and JKN. The following data were extracted from the eligible studies: authors’ names, journal, year of publication, gender, mean age, sample size, indications for surgery, surgical related information, inclusion and exclusion criteria, 24-h postoperative opioid consumption, postoperative pain scores at rest (24 h), PONV, complications and operative time.

### Data analysis

The extracted data were entered into the Review Manager software (RevMan, version 5.3; The Nordic Cochrane Centre, The Cochrane Collaboration, Copenhagen, 2012) for statistical analysis. For dichotomous outcomes, specifically PONV, the odds ratio (OR) was calculated along with its variance and 95% confidence interval (CI). For continuous outcomes, including pain scores, operative time, and opioid consumption, the standardized mean difference (SMD) with its corresponding 95% CI was calculated to compare the two intervention groups. All pooled effect estimates were derived using the random effects model as described previously by DerSimonian and Laird (10). The results for each outcome were graphically presented in forest plots displaying 95% CIs.

### Quality assessment

Using the Cochrane Systematic Review Handbook 5.1 and its recommended risk-of-bias assessment methodology, the quality of the included studies was thoroughly assessed. Various factors, such as the randomness of sequence generation, allocation concealment, blinding of trial participants and personnel, blinding of outcome assessors, incomplete outcome data, selective reporting, and other potential biases, were examined. The outcomes of the bias assessment were categorized as “low risk,” “high risk,” and “unclear.” Two researchers independently evaluated the study quality, and any conflicts were resolved t hrough mediation by the corresponding author.

## Results

### Study selection

**FIGURE 1:**
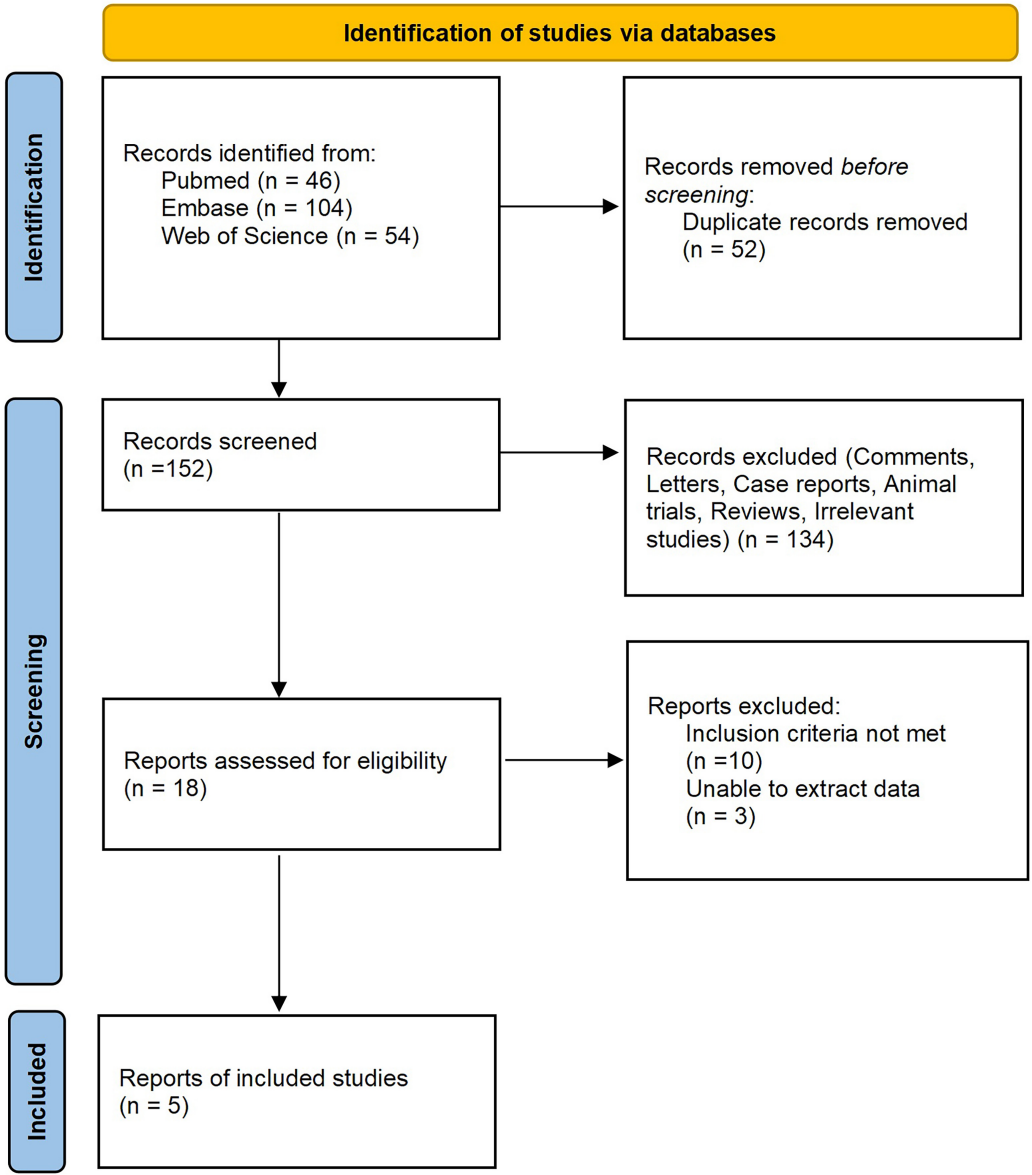
PRISMA flow diagram.

A total of 204 studies were identified from the initial search. After the removal of duplicates, 152 studies were screened. Subsequently, 18 studies were evaluated based on their full text, of which five studies (11–15) met the inclusion criteria for this meta-analysis. A PRISMA flow diagram summarizing the selection process is presented in Figure 1.

### Characteristics of the included studies

The characteristics of the five included studies are concisely summarized in Tables 1, 2. These studies were published between 2017 and 2024 and included a total of 585 patients, of whom 290 patients received Lap-TAPB and 295 patients underwent US-TAPB. Patient demographics across these studies were generally comparable, with no significant differences in age or sex distribution.

**TABLE 1:** Baseline characteristics of included RCTs.

Study	Study design	Lap-TAPB group			US-TAPB group			Pain scale
		No.	Age	Sex (male/female)	No.	Age	Sex (male/female)	
La Regina et al. (11)	Double-blind, RCT	55	65.7 (12.6)	31/24	57	68.1 (12.8)	17/40	VAS
Park et al. (12)	Single-blind, RCT	38	62.0 ± 10.6	29/9	35	62.2 ± 7.5	23/12	NRS
Wong et al. (15)	Single-blind, RCT	29	61.5 ± 14.3	19/10	31	60.0 ± 13.6	16/15	PRS
Zahiyan et al. (13)	Single-blind, RCT	41	48.4 ± 18.8	16/25	45	54.0 ± 16.6	22/23	VAS
Salmonsen et al. (14)	Single-blind, RCT	127	73.8	80/47	127	72.7	64/63	NRS

Lap-TAPB, laparoscopic-guided transversus abdominis plane block; US-TAPB, ultrasound-guided transversus abdominis plane block; VAS, visual analog scale; NRS, numerical rating scale; PRS, patient reported pain score.

**TABLE 2:** Baseline characteristics of included RCTs.

Study	Indication for surgery	Surgical approach	ASA	Specimen extraction site	Timing of block	Location of block	TAP block compasition and volume
La Regina et al. (11)	CRC, diverticular disease, polyps and IBD	Laparoscopic	Not reported	Midline	Before surgery	The anterior axillary line midway between the iliac crest and the costal margin	15 mL of 0.2% ropivacaine (30 mL in total)
Park et al. (12)	CRC	Laparoscopic	1–2	Midline	Before surgery	At the midaxillary line between the lower costal margin and the iliac crest	30 mL of 0.25% ropivacaine (60 mL in total)
Wong et al. (15)	CRC and benign cases	Laparoscopic	Not reported	Midline	At end of surgery	The lateral border of the rectus and inferior to the costal margin	0.25% bupivacaine with 1:100,000 epinephrine with a volume of 1 cc/kg (up to 60 cc)
Zahiyan et al. (13)	CRC and IBD	Laparoscopic and robotic	1–3	Pfannenstiel incision	At end of surgery	At the anterior axillary line between the costal margin and iliac crest	1 mL/kg total of 50:50 mix of 0.25% bupivacaine with epinephrine 1:200,000 and 0.25% bupivacaine without epinephrine
Salmonsen et al. (14)	CRC and adenoma	Laparoscopic	1–4	Not reported	Before surgery	A dual subcostal approach.	20 mL of 2 mg/mL ropivacaine (40 mL in total)

CRC, colorectal cancer; IBD, inflammatory bowel disease; ASA, American Society of Anesthesiologists.

### Risk-of-bias assessment

**FIGURE 2:**
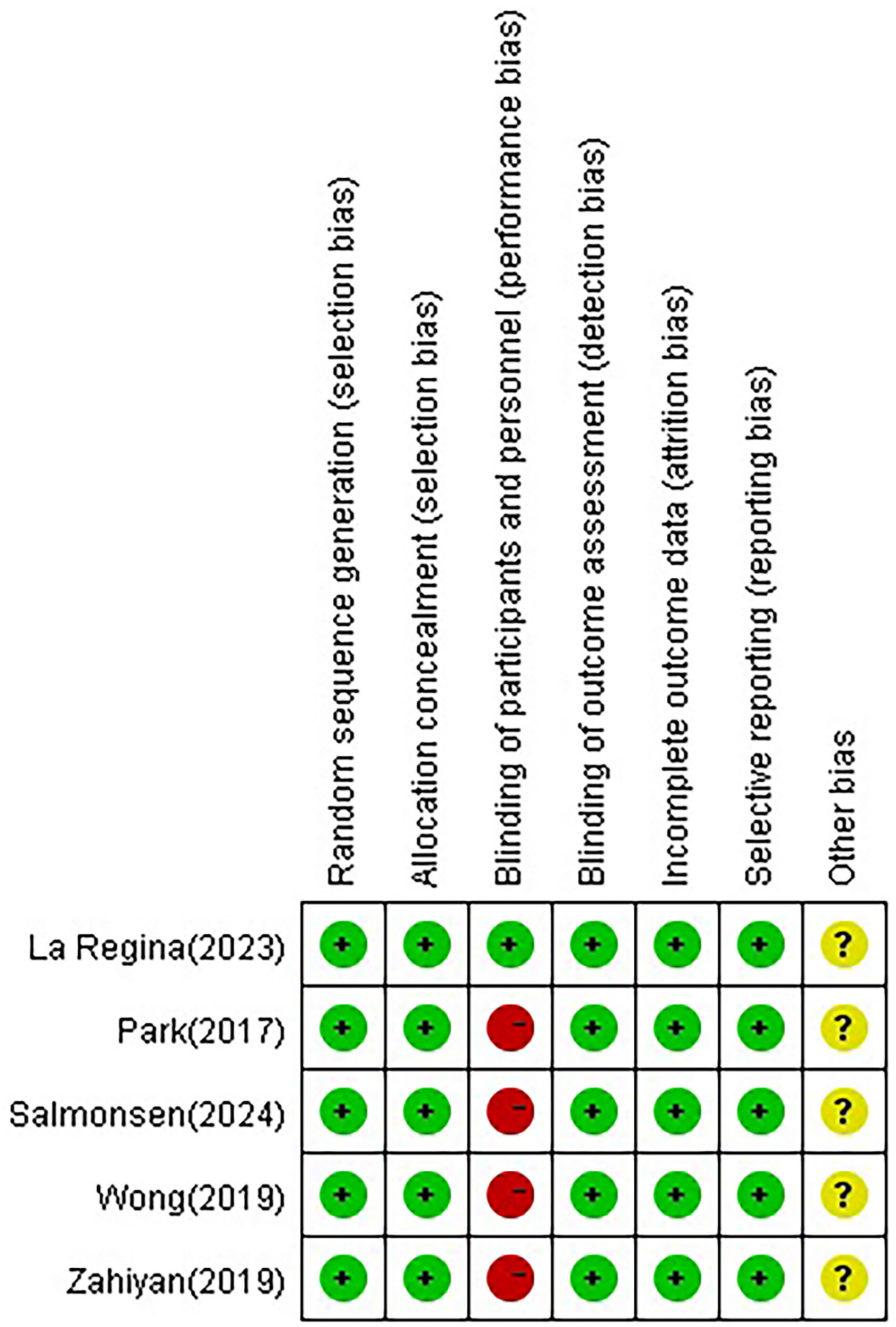
Risk of bias summary.

After conducting a quality assessment on the included studies using the Cochrane Risk of Bias Assessment Tool, the overall quality of the entire literature was considered good (Figure 2).

### Primary outcome: opioid consumption at 24 h

**FIGURE 3:**
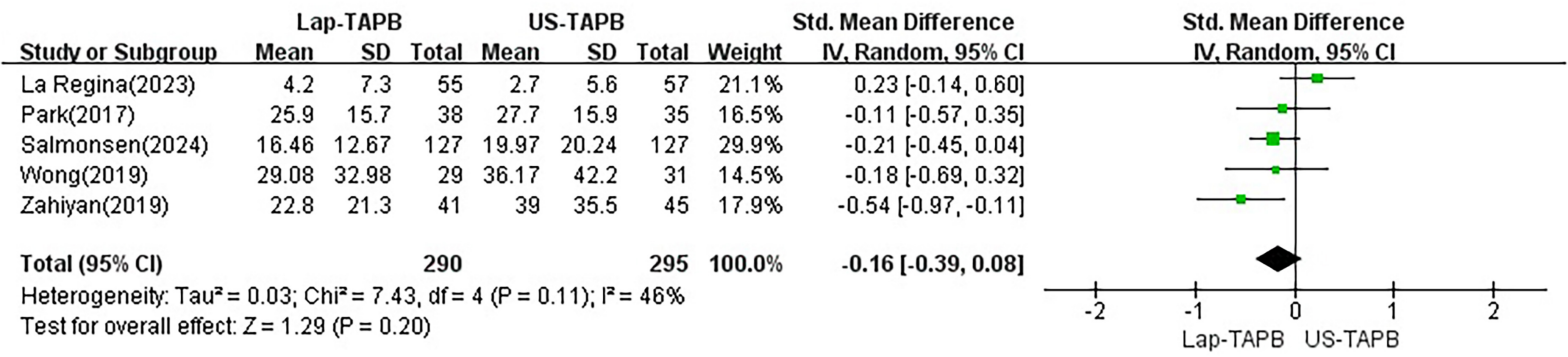
Meta-analysis of opioid consumption at 24 h.

Data on 24-h postoperative opioid consumption were available in all five studies (11–15). Random effects analysis revealed that Lap-TAPB was not associated with significantly lower postoperative opioid consumption at 24 h compared to US-TAPB [SMD −0.16, 95% CI = −0.39 to 0.08, *p* = 0.20; chi^2^ = 7.43 (df = 4), *p* = 0.11, *I*^2^ = 46%] (Figure 3).

### Secondary outcomes

#### Postoperative pain scores at rest (24 h)

**FIGURE 4:**
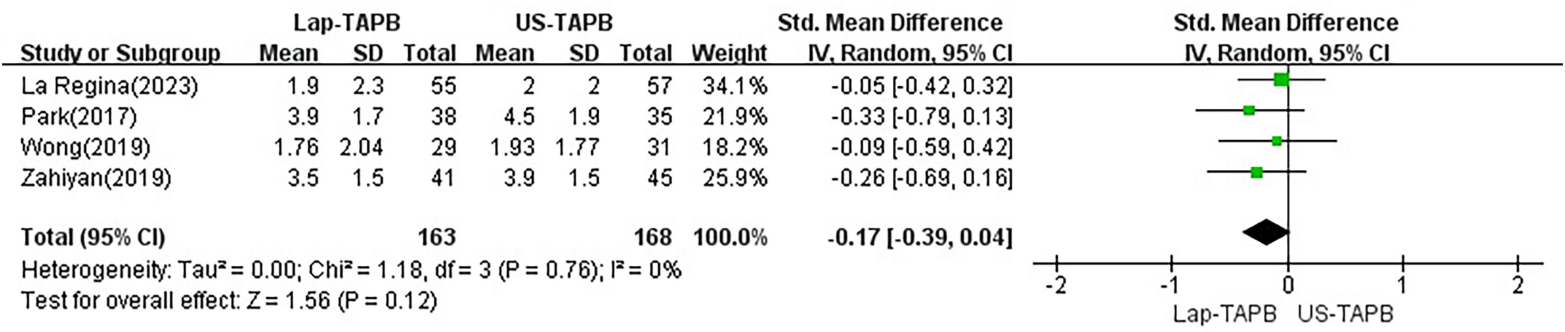
Meta-analysis of pain scores at rest at 24 h.

Postoperative pain scores at rest (24 h) were reported by four studies (11–13, 15). Random effects analysis showed that Lap-TAPB did not result in a significantly lower pain score compared with US-TAPB [SMD −0.16, 95% CI = −0.39 to 0.08, *p* = 0.20; chi^2^ = 7.43 (df = 4), *p* = 0.11, *I*^2^ = 46%] (Figure 4).

#### Mean operative time

**FIGURE 5:**
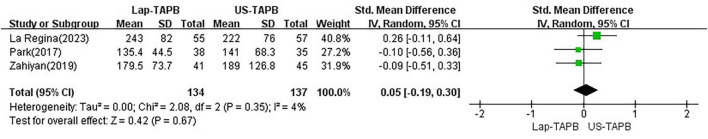
Meta-analysis of operative time.

Data on operative time were available in three studies, which included a total of 271 patients (11–13). On random effects analysis, no significant difference was observed between the groups [SMD 0.05, 95% CI = −0.19 to 0.30, *p* = 0.67; chi^2^ = 2.08 (df = 2), *p* = 0.35, *I*^2^ = 4%] (Figure 5).

#### PONV

**FIGURE 6:**
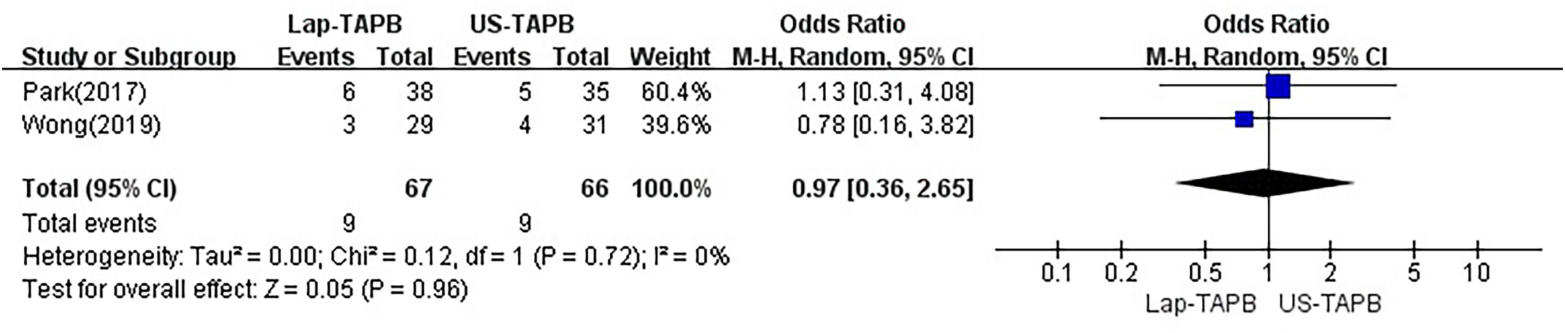
Meta-analysis of PONV.

Data on PONV were reported in two studies, which included a total of 133 patients (12, 15). Random effects analysis revealed no significant difference between the groups [OR = 0.97, 95% CI = 0.36–2.65, *p* = 0.96; chi^2^ = 0.12 (df = 1), *p* = 0.72, *I*^2^ = 0%] (Figure 6).

#### Complications

**FIGURE 7:**
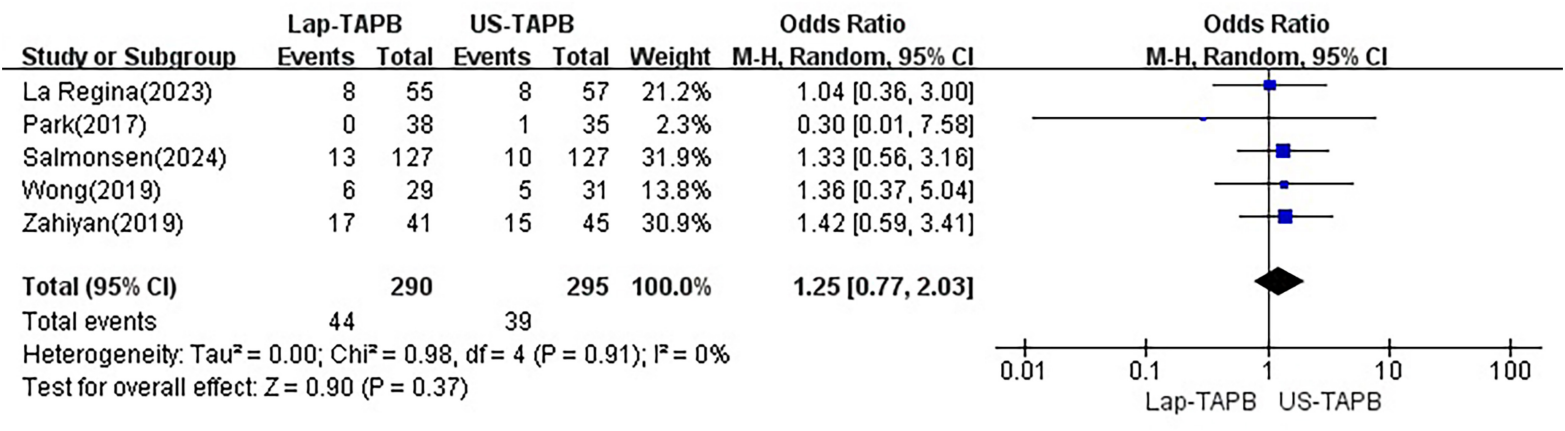
Meta-analysis of complications.

Data on complications were reported in five studies, which included a total of 585 patients (11–15). Random effects analysis revealed no significant difference between the groups [OR = 1.25, 95% CI = 0.77–2.03, *p* = 0.37; chi^2^ = 0.98 (df = 4), *p* = 0.91, *I*^2^ = 0%] (Figure 7).

## Publication bias

Consistent with the guidelines stipulated in the Cochrane Handbook for Systematic Reviews of Interventions, analysis of publication bias was deemed unnecessary as none of the study groups comprised > 10 studies.

## Discussion

Our updated systematic review and meta-analysis, incorporating the latest high-quality evidence, including the multicenter noninferiority trial by La Regina et al. (11), demonstrates that these two approaches are clinically equivalent in terms of five critical outcomes: 24-h postoperative opioid consumption, postoperative pain scores at rest (24 h), PONV incidence, operative time and complications.

This finding represents a significant advancement from earlier meta-analytic findings. Copperthwaite et al. (8), in their 2022 review of three small RCTs (*n* = 219), reported statistically significant reductions in opioid consumption (SMD 0.30, 95% CI = −0.57 to −0.03, *p* = = 0.03) and pain scores (SMD 0.29, 95% CI = −0.56 to −0.03, *p* = 0.03) at 24 h with L-TAP. However, those studies suffered from methodological limitations, including a single-center design, lack of blinding, and heterogeneous analgesic regimens, which may have inflated the effect sizes. The subsequent publication of La Regina et al.’s (11) rigorously designed trial considerably alters the evidence landscape. In their study, the median 24-h opioid consumption was nearly identical between the groups, and postoperative pain scores at rest (24 h) exhibited no significant difference. When pooled with prior data using a random effects model, our analysis confirmed statistical and clinical equivalence across primary outcomes. Also, no significant differences were observed in pain scores at rest at 24 h (*p* = 0.12), incidence of PONV (*p* = 0.96), operative time (*p* = 0.67), and complications (*p* = 0.37).

However, acute postoperative pain remains the most common concern of ERAS. Meanwhile, regular opioid administration is associated with postoperative ileus, PONV, delayed mobilization, acute urinary retention, and early-term somnolence and delirium (16). In the context of highly recommended laparoscopic surgery, it is easy to overlook a serious issue of postoperative pain, which is related to factors such as the acidic abdominal environment during surgery, the volume of insufflated gas, residual abdominal gas after laparoscopic surgery, wound size, the presence of drainage tubes, and anesthesia drugs. Therefore, up to 80% of patients require opioid analgesia after surgery (17). TAPB is widely employed during laparoscopic colorectal surgery and has proven to be effective in reducing postoperative opioid consumption (18). In the 2018 ERAS Society Guidelines for Perioperative Care in Elective Colorectal Surgery, the use of TAPB instead of epidural analgesia is strongly recommended in colorectal minimally invasive surgery (19). Lap-TAPB and US-TAPB have reduced the risk of peritoneal penetration and facilitated the accurate identification of the tissue plane (20). Our study showed that both delivery methods of the TAP block were effective in postoperative pain control. Meanwhile, there was no difference in operative time, postoperative PONV incidence, and complications between the two different methods. Therefore, for patients undergoing minimally invasive colorectal surgery, choosing either method can achieve postoperative pain relief.

Marked by its capacity for dynamic maneuvers and evaluation of long nerve segments, no radiation exposure and a few contraindications, as well as portability, ultrasonography is recognized as one of the optimal imaging modalities for peripheral nerves (21), which contributes to its widespread application in perioperative nerve blocks. Conversely, owing to additional human resources, time, and economic costs, ultrasound-guided nerve block techniques might not be available in primary hospitals. Furthermore, despite the guidance of ultrasound, procedure-related inadvertent visceral injury should not be ignored (22). Laparoscopic visualization minimizes intraperitoneal injection and visceral injury originating from peritoneal penetration and can be a potential alternative to reduce the unnecessary use of healthcare resources.

This study has several limitations that need to be acknowledged. First, most trials evaluated pain mainly at rest; dynamic pain (e.g., during coughing or mobilization) appears to be more sensitive to the effects of TAP but was inconsistently reported. Second, long-term outcomes, such as chronic postsurgical pain or functional recovery beyond 48 h, remain unexplored. Third, cost-effectiveness analyses comparing L-TAP and US-TAP are lacking, despite implications for staffing, equipment, and throughput. Fourth, although this study is an updated meta-analysis, the inclusion of only five studies compromises the statistical power of subgroup analyses and limits the robustness of the conclusions.

In summary, the current highest-level evidence indicates that laparoscopic and ultrasound-guided TAP blocks provide comparable analgesia, safety, and procedural efficiency in laparoscopic colorectal surgery. Therefore, the choice between techniques should be individualized based on team expertise, institutional resources, and integration into existing ERAS workflows, not anticipated differences in core clinical outcomes. Future research should prioritize patient-centered endpoints, economic evaluations, and standardization of block protocols (e.g., volume, concentration, and compartment targeted) to optimize the role of TAP in colorectal surgical care.

## Data Availability

The original contributions presented in this study are included in the article/supplementary material, further inquiries can be directed to the corresponding author.

## References

[1] Irani J Hedrick T Miller T Lee L Steinhagen E Shogan B et al. Clinical practice guidelines for enhanced recovery after colon and rectal surgery from the American society of colon and rectal surgeons and the society of American gastrointestinal and endoscopic surgeons. *Dis Colon Rectum.* (2023) 66:15–40. 10.1097/DCR.0000000000002650 36515513 PMC9746347

[2] Ljungqvist O Scott M Fearon K. Enhanced recovery after surgery: a review. *JAMA Surg.* (2017) 152:292–8. 10.1001/jamasurg.2016.4952 28097305

[3] Rafi A. Abdominal field block: a new approach via the lumbar triangle. *Anaesthesia.* (2001) 56:1024–6. 10.1046/j.1365-2044.2001.02279-40.x 11576144

[4] McDonnell J O’Donnell B Curley G Heffernan A Power C Laffey J. The analgesic efficacy of transversus abdominis plane block after abdominal surgery: a prospective randomized controlled trial. *Anesth Analg.* (2007) 104:193–7. 10.1213/01.ane.0000250223.49963.0f 17179269

[5] Haruethaivijitchock P Ng J Taksavanitcha G Rattananupong T Lohsoonthorn V Sahakitrungruang C et al. Postoperative analgesic efficacy of modified continuous transversus abdominis plane block in laparoscopic colorectal surgery: a triple-blind randomized controlled trial. *Tech Coloproctol.* (2020) 24:1179–87. 10.1007/s10151-020-02311-9 32725352

[6] Tikuisis R Miliauskas P Lukoseviciene V Samalavicius N Dulskas A Zabuliene L et al. Transversus abdominis plane block for postoperative pain relief after hand-assisted laparoscopic colon surgery: a randomized, placebo-controlled clinical trial. *Tech Coloproctol.* (2016) 20:835–44. 10.1007/s10151-016-1550-3 27896461

[7] Zhao Y Zhang H Yuan Z Han Y Chen Y Liu Q et al. Analgesic efficacy of postoperative bilateral, ultrasound-guided, posterior transversus abdominis plane block for laparoscopic colorectal cancer surgery: a randomized, prospective, controlled study. *BMC Anesthesiol.* (2021) 21:107. 10.1186/s12871-021-01317-6 33823786 PMC8022542

[8] Copperthwaite A Sahebally S Raza Z Devane L McCawley N Kearney D et al. A meta-analysis of laparoscopic versus ultrasound-guided transversus abdominis plane block in laparoscopic colorectal surgery. *Ir J Med Sci.* (2023) 192:795–803. 10.1007/s11845-022-03017-7 35499808

[9] Liberati A Altman D Tetzlaff J Mulrow C Gøtzsche P Ioannidis J et al. The PRISMA statement for reporting systematic reviews and meta-analyses of studies that evaluate health care interventions: explanation and elaboration. *PLoS Med.* (2009) 6:e1000100. 10.1371/journal.pmed.1000100 19621070 PMC2707010

[10] DerSimonian R Laird N. Meta-analysis in clinical trials revisited. *Contemp Clin Trials.* (2015) 45:139–45. 10.1016/j.cct.2015.09.002 26343745 PMC4639420

[11] La Regina D Popeskou S Saporito A Gaffuri P Tasciotti E Dossi R et al. Laparoscopic versus ultrasound-guided transversus abdominis plane block in colorectal surgery: a non-inferiority, multicentric randomized double-blinded clinical trial. *Colorectal Dis.* (2023) 25:1921–8. 10.1111/codi.16689 37525414

[12] Park S Park J Choi G Kim H Moon S Yeo J. Comparison of analgesic efficacy of laparoscope-assisted and ultrasound-guided transversus abdominis plane block after laparoscopic colorectal operation: a randomized, single-blind, non-inferiority trial. *J Am Coll Surg.* (2017) 225:403–10. 10.1016/j.jamcollsurg.2017.05.017 28610880

[13] Zaghiyan K Mendelson B Eng M Ovsepyan G Mirocha J Fleshner P. Randomized clinical trial comparing laparoscopic versus ultrasound-guided transversus abdominis plane block in minimally invasive colorectal surgery. *Dis Colon Rectum.* (2019) 62:203–10. 10.1097/DCR.0000000000001292 30540660

[14] Salmonsen C Lange K Kleif J Krøijer R Bruun L Mikalonis M et al. Transversus abdominis plane block in minimally invasive colon surgery: a multicenter three-arm randomized controlled superiority and non-inferiority clinical trial. *Reg Anesth Pain Med.* (2025): Online ahead of print. 10.1136/rapm-2024-105712 39542642 PMC13151517

[15] Wong D Curran T Poylin V Cataldo T. Surgeon-delivered laparoscopic transversus abdominis plane blocks are non-inferior to anesthesia-delivered ultrasound-guided transversus abdominis plane blocks: a blinded, randomized non-inferiority trial. *Surg Endosc.* (2020) 34:3011–9. 10.1007/s00464-019-07097-y 31485929 PMC7103091

[16] McEvoy M Scott M Gordon D Grant S Thacker J Wu C et al. American society for enhanced recovery (ASER) and Perioperative quality initiative (POQI) joint consensus statement on optimal analgesia within an enhanced recovery pathway for colorectal surgery: part 1-from the preoperative period to PACU. *Perioper Med.* (2017) 6:8. 10.1186/s13741-017-0064-5 28413629 PMC5390366

[17] Mouton W Bessell J Otten K Maddern G. Pain after laparoscopy. *Surg Endosc.* (1999) 13:445–8. 10.1007/s004649901011 10227938

[18] Mukhtar K Singh S. Transversus abdominis plane block for laparoscopic surgery. *Br J Anaesth.* (2009) 102:143–4. 10.1093/bja/aen338 19059927

[19] Gustafsson U Scott M Hubner M Nygren J Demartines N Francis N et al. Guidelines for perioperative care in elective colorectal surgery: enhanced recovery after surgery (ERAS§) society recommendations: 2018. *World J Surg.* (2019) 43:659–95. 10.1007/s00268-018-4844-y 30426190

[20] Hamid H Emile S Saber A Ruiz-Tovar J Minas V Cataldo T. Laparoscopic-guided transversus abdominis plane block for postoperative pain management in minimally invasive surgery: systematic review and meta-analysis. *J Am Coll Surg.* (2020) 231:376–86.e15. 10.1016/j.jamcollsurg.2020.05.020 32502615

[21] Ali Z Pisapia J Ma T Zager E Heuer G Khoury V. Ultrasonographic evaluation of peripheral nerves. *World Neurosurg.* (2016) 85:333–9. 10.1016/j.wneu.2015.10.005 26463397

[22] Lancaster P Chadwick M. Liver trauma secondary to ultrasound-guided transversus abdominis plane block. *Br J Anaesth.* (2010) 104:509–10. 10.1093/bja/aeq046 20228188

